# Supplementary material for: The interactome of intact mitochondria by cross-linking mass spectrometry provides evidence for coexisting respiratory supercomplexes
Source: Mol Cell Proteomics. 2017 Dec 8;17(2):216–32. doi: 10.1074/mcp.RA117.000470 (PMC5795388; doi:10.1074/mcp.RA117.000470)
Supplement: Supplemental Data [file supp_RA117.000470_133922_0_supp_23978_fzffwf.zip › spectra_annotation/mito_spectra_annotation/486-1-12-1-12-1.pdf]

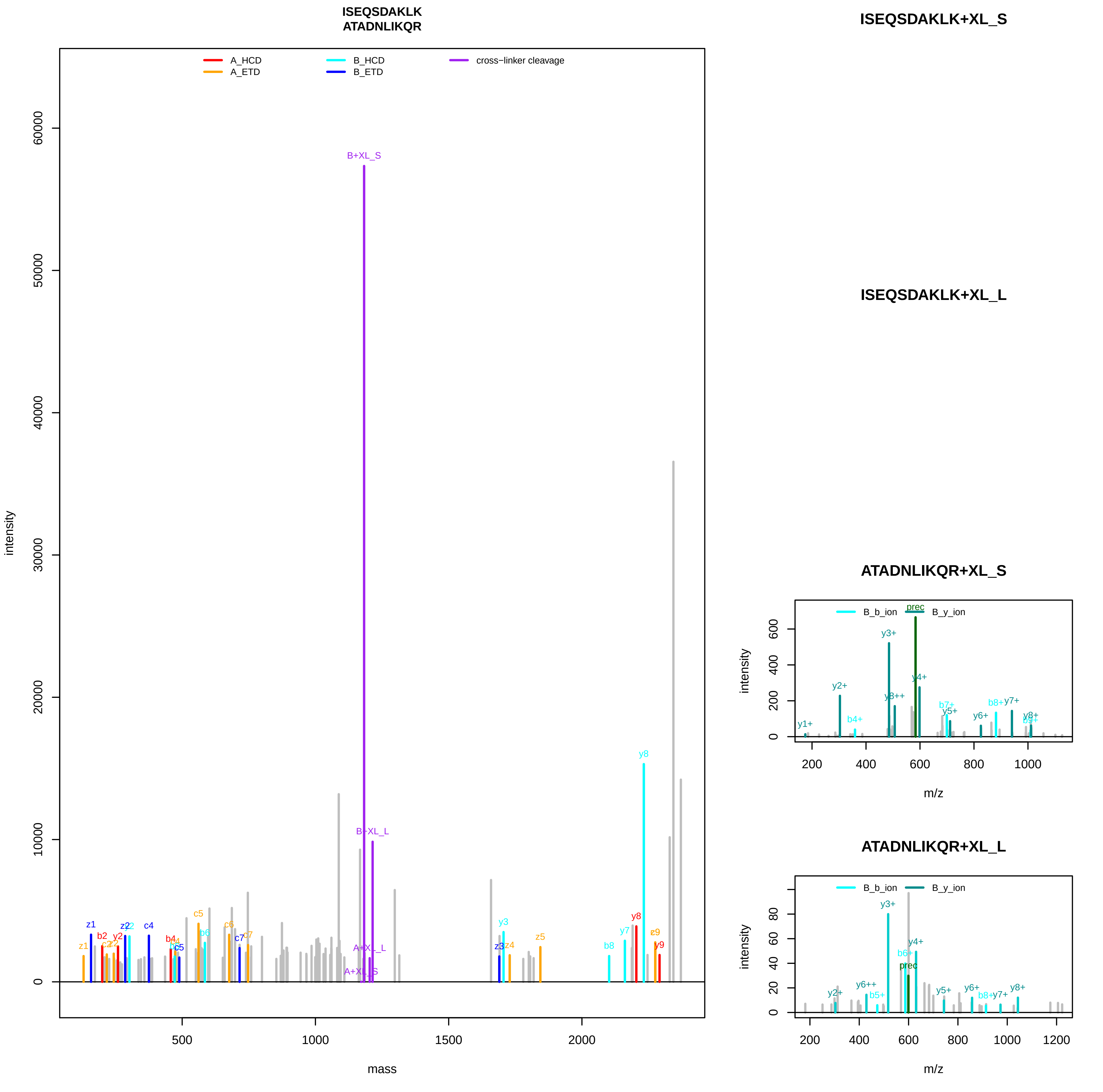

ISEQSDAKLK
ATADNLIKQR
A_HCD
A_ETD
B_HCD
B_ETD
cross−linker cleavage
intensity
mass
B+XL_S
B+XL_L
A+XL_L
A+XL_S
y8
y7
y9
b8
y3
z3
z4
z5
z9
c5
c6
c7
b6
b4
c4
c5
z1
b2
y2
z2
ISEQSDAKLK+XL_S
ISEQSDAKLK+XL_L
ATADNLIKQR+XL_S
B_b_ion
B_y_ion
prec
y3+
y2+
y4+
y8++
b4+
y1+
b7+
y5+
b8+
y7+
y6+
y8+
b9+
m/z
ATADNLIKQR+XL_L
y6++
b5+
b6+
y5+
y6+
b8+
y7+
y8+
